# Inferior gluteal artery pseudoaneurysm after fall from a bicycle: case report

**DOI:** 10.1590/1677-5449.003018

**Published:** 2018

**Authors:** Renato Fanchiotti Costa, Ricardo de Alvarenga Yoshida, Rodrigo Jaldin Gibin, Marcone Lima Sobreira, Rafael Elias Fares Pimenta, Matheus Bertanha, Paula Angeleli Bueno de Camargo, Winston Bonetti Yoshida

**Affiliations:** 1 Clínica Angiovalle, São José dos Campos, SP, Brasil.; 2 Universidade Estadual Paulista – UNESP, Faculdade de Medicina de Botucatu, Departamento de Cirurgia e Ortopedia, Botucatu, SP, Brasil.

**Keywords:** buttocks/blood supply, aneurysm, false, wounds and injury, accidents, traffic

## Abstract

Pseudoaneurysms of gluteal arteries are rare, especially involving the inferior gluteal artery. They are mainly associated with penetrating trauma, infections, or pelvic fractures. A minority of cases are caused by blunt traumas, with only six cases reported in English. We present a case of pseudoaneurysm of the right inferior gluteal artery after a bicycle fall, presenting with a large hematoma in the gluteal region, observed during clinical examination, and significantly reduced hemoglobin. CT angiography revealed a large hematoma, with contrast extravasation and pseudoaneurysm formation. Angiography revealed that the origin of the lesion was in the right inferior gluteal artery. This artery was embolized with coils. After the procedure, the patient was referred to an intensive care unit, from where he was later transferred to a different hospital, with bleeding controlled. Endovascular treatment of these cases is a safe, fast and an effective option.

## INTRODUCTION

 Aneurysms of the gluteal arteries are rare conditions, accounting for less than 1% of all aneurysms. 1 ^-^ 3 The superior and inferior gluteal arteries are involved more frequently, while aneurysms of the persistent sciatic artery are less common. With these arteries, pseudoaneurysms are more common than true aneurysms, and frequency is greatest in the superior gluteal artery. 1

 Reports published during the last 30 years describe pseudoaneurysms of the inferior gluteal artery caused by penetrating traumas, fractures of bones of the pelvis, or iatrogenic injuries during surgical procedures involving the pelvis or hips. 1 Pseudoaneurysms of the inferior gluteal artery after blunt trauma are even rarer, with just six cases reported in the literature. 3

 This article describes a case of inferior gluteal artery pseudoaneurysm after a blunt trauma (due to falling off a bicycle onto a stone, causing the blunt gluteal trauma). It illustrates the importance of considering an arterial injury in the gluteal region even after blunt trauma, in addition to describing endovascular treatment of the condition. 

## CASE DESCRIPTION

**Figure 1:**
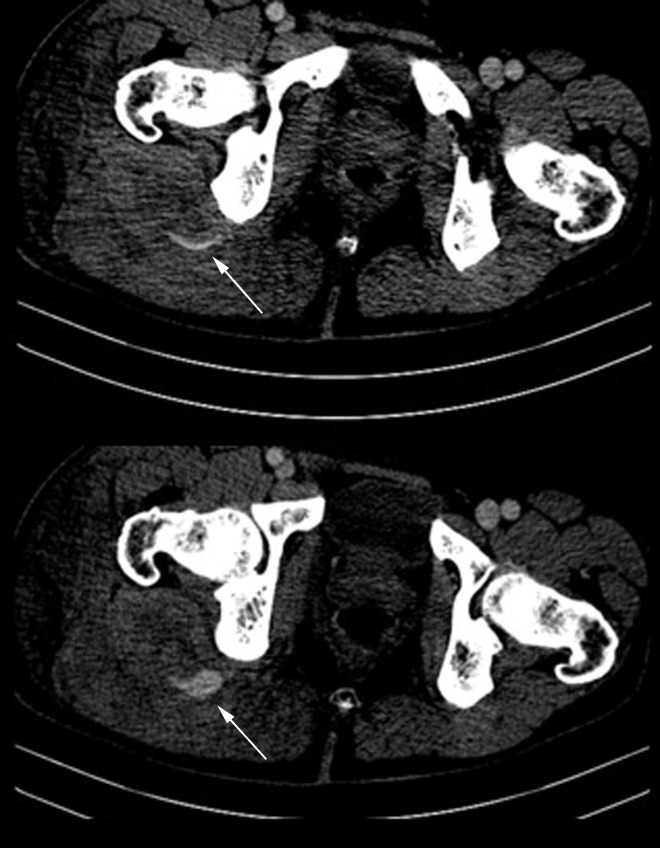
The arrow indicates the lesion in the tomographic image, with leakage of contrast in the right gluteal region and the associated hematoma.

**Figure 2:**
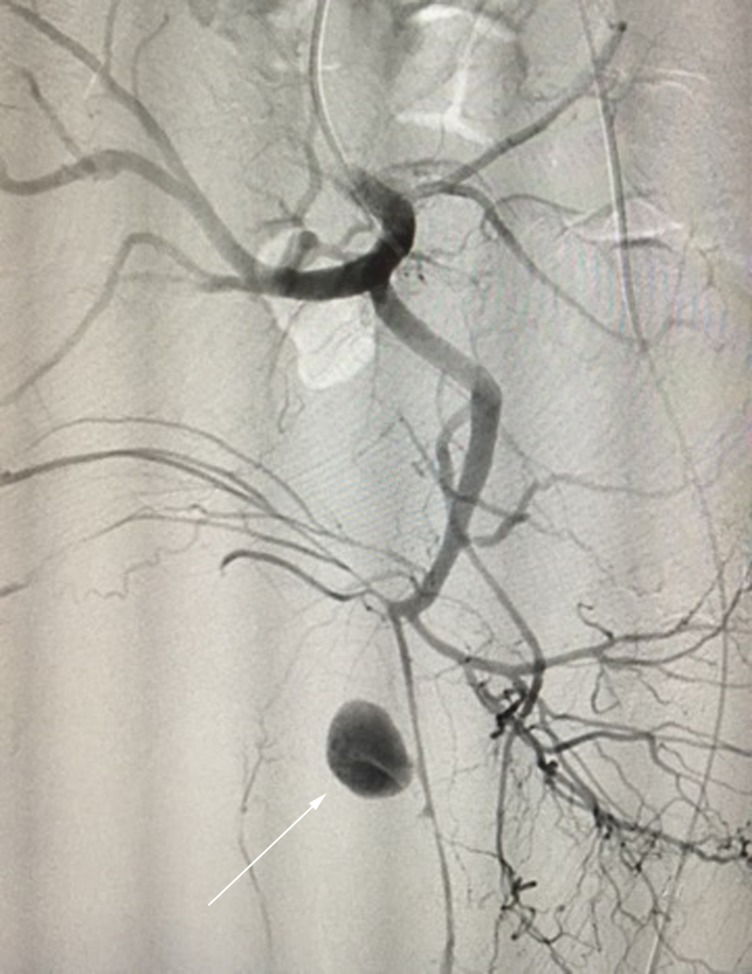
Angiographic image with arrow indicating a pseudoaneurysm of the right inferior gluteal artery.

 A male, 57-year-old patient collided with another competitor during a bicycle race and fell off, landing with his right gluteus hitting a stone. He presented at an emergency room with considerable pain and edema in the right gluteal region. According to the Advanced Trauma Life Support (ATLS) protocol, the patient’s airway was clear, breathing was normal, he showed signs of class II shock (heart rate greater than 100 beats per minute), scored 15 on the Glasgow Coma Scale, and was free from signs of pelvic bone instability. During physical examination, a significant expanse of non-pulsating hematoma was noted in the right lumbar and gluteal region, painful on local palpation. The patient also complained of right foot paresthesia, probably caused by compression of the sciatic nerve. Laboratory test results showed reduced hemoglobin (< 7 g/dL), and replacement was initiated with packed red blood cells. An angiotomography of the abdomen and pelvis was performed, showing hematoma of the right gluteus, with contrast leakage, compatible with a pseudoaneurysm at that site ( Figure 1 ). The patient was taken to a hemodynamic suite and underwent angiography, which confirmed a pseudoaneurysm of the inferior gluteal artery ( Figure 2 ). 

**Figure 3:**
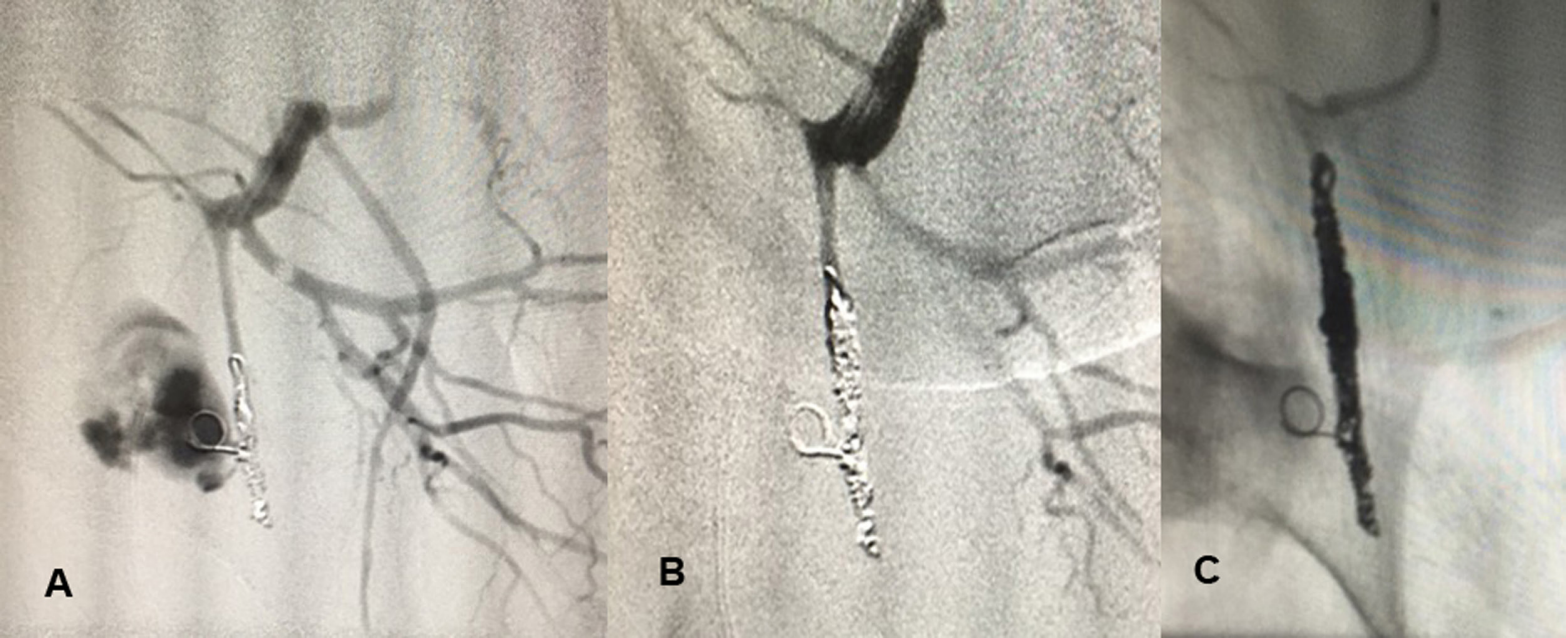
(A) Intraoperative angiographic image showing the patent pseudoaneurysm; (B and C) Final angiographic appearance after coil embolization, showing complete occlusion of the pseudoaneurysm.

 Superselective embolization was performed to repair the pseudoaneurysm, occluding the branch involved proximal and distal of the lesion using controlled-release coils (Codman & Shurtleff, Inc. brand; by Johnson & Johnson, Raynham, MA, United States) to completely stop the bleeding, during the same procedure as angiography ( Figures 3 3C). After embolization, the hematoma was drained to reduce the risk of gluteal necrosis, relieve pain, and improve neurological signs and symptoms. The patient was transferred to the intensive care unit, where hemodynamic and laboratory parameters were monitored and volume resuscitation was supplemented. There was immediate improvement of gluteal pain and paresthesia. The patient was later transferred to another hospital, with bleeding controlled. 

## DISCUSSION

 The most important arteries supplying the gluteal region are the superior and inferior gluteal arteries. The inferior gluteal artery is the largest terminal branch of the anterior segment of the internal iliac artery. A pseudoaneurysm is caused by a rupture of the vessel wall and presents as a pulsating hematoma that communicates with the artery. Normally, the branches of this artery irrigate a greater proportion of the gluteus than the branches of the superior gluteal artery. 4 When a person falls onto the ground or other rigid surface, suffering a blunt trauma, the inferior gluteal artery and its branches are more likely to be injured than the superior gluteal artery, because of the paths they take through the gluteal region. 1 ^,^ 3

 Fewer than 150 cases of pseudoaneurysms of the gluteal artery have been described in the literature to date. 5 These injuries more commonly involve the superior gluteal artery and are related to penetrating traumas, iatrogenic injuries during pelvic or hip surgery, or fractures of pelvic bones, or occur after intramuscular injections. 2 ^,^ 4 They may occur soon after the initial trauma or weeks, months, or years after the event. 3 Less frequently, they may be caused by blunt traumas, as described here. 

 Pseudoaneurysms may be asymptomatic, but the most common presentation is with edema and/or painful hematoma in the gluteal region. Since symptoms are nonspecific, there is a need to conduct differential diagnosis to rule out gluteal abscesses or pain of sciatic origin. 4 The most common signs are pulsating hematoma and thrill in the gluteal region affected, which may be accompanied by local inflammation or symptoms of sciatic nerve compression. 3 ^,^ 4 They may present with hemorrhagic shock, when the pseudoaneurysm ruptures, and this is the most severe form of presentation. An incorrect diagnosis of a local abscess can have catastrophic consequences. 1 ^,^ 3 ^,^ 4 In the case described here, the signs observed were painful, non-pulsating hematoma of the right gluteal region. 

 Since there is a risk of a potentially fatal situation, early diagnosis and immediate treatment are of fundamental importance. Angiotomography is highly accurate for diagnosing pseudoaneurysms and for differentiating them from abscesses or soft tissue tumors. 1 ^,^ 5 ^,^ 6 In the present case, the patient underwent angiotomography because he was hemodynamically stable. Ultrasonography with Doppler may be the first work-up examination to be used when the patient first presents because it is low cost and noninvasive and can show arterial flow within the hematoma interior and can differentiate other types of soft tissue injuries. 1 ^,^ 5

 While angiography can be used both to confirm the pseudoaneurysm site and, if feasible, conduct endovascular treatment immediately, gluteal artery injuries can be treated either with conventional surgical approaches (Battle) or with minimally invasive endovascular techniques such as transcatheter embolization. 7 ^,^ 8 The advantages of embolization include: absence of scars, reduced infection risk, preservation of the retroperitoneal space, lower risk of iatrogenic injuries to nerves or vessels, and shorter length of hospital stay. 9 ^,^ 10 In this type of case, superselective embolization is a safe, effective, and reliable method for stopping arterial bleeding, especially in the pelvic region. 2 ^,^ 11 ^,^ 12 In the present case, emergency embolization successfully stopped the bleeding, avoiding potential permanent damage to the sciatic nerve by compression caused by the expanding gluteal hematoma. The potential risk of this type of procedure (embolization) is ischemia of the gluteal muscle. 2 ^,^ 13 ^,^ 14 Superselective embolization with controlled release coils reduces this risk. 1 ^,^ 11 Alternatives to coil embolization include embolization with glue or other emboligenic agents, such as cyanoacrylate glue and onyx. 14

 Some authors also recommend ultrasound-guided injections of thrombin into the lesion site as an alternative to coil embolization. This is a treatment method widely used with superficial and narrow-necked pseudoaneurysms, such as injuries to the common femoral artery after angiography. 1 ^,^ 15 The risk of this procedure is possible reflux of thrombin into the arterial circulation, causing severe thrombosis of the vessels surrounding the lesion. 4 In this case, the pseudoaneurysm of the gluteal artery was deep and surrounded by the hematoma, making puncture for direct injection difficult. 

## CONCLUSIONS

 Pseudoaneurysm of the inferior gluteal artery is still a rare and difficult to diagnosis condition that challenges both trauma surgeons and vascular surgeons. Endovascular treatment is a feasible, safe, and effective option and should be the first-line choice when possible, because of the lower invasivity compared to conventional treatment. 
