# Supplementary material for: Inferior gluteal artery pseudoaneurysm after fall from a bicycle: case report
Source: J Vasc Bras. 2018 Oct-Dec;17(4):353–7. doi: 10.1590/1677-5449.003018 (PMC6375272; doi:10.1590/1677-5449.003018)
Supplement: Translation

# Translation: Pseudoaneurisma da artéria glútea inferior após queda de bicicleta: relato de caso

**Authors:** Renato Fanchiotti Costa, Ricardo de Alvarenga Yoshida, Rodrigo Jaldin Gibin, Marcone Lima Sobreira, Rafael Elias Fares Pimenta, Matheus Bertanha, Paula Angeleli Bueno de Camargo, Winston Bonetti Yoshida

**Affiliations:** 1 Clínica Angiovalle, São José dos Campos, SP, Brasil.; 2 Universidade Estadual Paulista – UNESP, Faculdade de Medicina de Botucatu, Departamento de Cirurgia e Ortopedia, Botucatu, SP, Brasil.

**Keywords:** nádegas/irrigação sanguínea, falso aneurisma, ferimentos e lesões, acidentes de trânsito

## Abstract

Pseudoaneurismas de artérias glúteas são raros, especialmente os que envolvem a artéria glútea inferior. Eles estão associados principalmente a traumas penetrantes, infecções ou fraturas de pelve. Em uma minoria de casos, são causados por traumas fechados, havendo somente seis casos relatados na literatura. Apresenta-se aqui um caso de pseudoaneurisma da artéria glútea inferior direita após queda de bicicleta, evoluindo com grande hematoma na região glútea ao exame clínico e queda hematimétrica significativa. A angiotomografia revelou um grande hematoma na região glútea, com extravasamento de contraste e formação de pseudoaneurisma no local. A angiografia revelou que a origem da lesão era na artéria glútea inferior direita. Foi realizada embolização dessa artéria com molas. Após esse procedimento, o paciente foi encaminhado para a unidade de terapia intensiva, de onde foi posteriormente transferido para outro hospital, com o sangramento controlado. Para esses casos, o tratamento endovascular é uma opção segura, rápida e efetiva.

## INTRODUÇÃO

 Aneurismas das artérias glúteas são condições raras, representando menos de 1% de todos os aneurismas 1 ^-^ 3 . As artérias glúteas superior e inferior são as mais acometidas e, mais raramente, a artéria isquiática persistente. Os pseudoaneurismas dessas artérias são mais comuns que os aneurismas verdadeiros, com mais frequência na artéria glútea superior 1 . 

 Nos relatos das últimas três décadas, os pseudoaneurismas da artéria glútea inferior foram causados por traumas penetrantes, fraturas de ossos da pelve ou lesões iatrogênicas durante procedimentos cirúrgicos da pelve ou do quadril 1 . Pseudoaneurismas da artéria glútea inferior após trauma fechado são ainda mais raros, havendo somente seis casos relatados na literatura 3 . 

 Relata-se aqui um caso de pseudoaneurisma da artéria glútea inferior após trauma fechado (queda de bicicleta com trauma contuso de glúteo contra uma pedra). Este artigo destaca a importância de se considerar uma lesão arterial na região glútea mesmo após um trauma fechado, além do tratamento endovascular dessa afecção. 

## DESCRIÇÃO DO CASO

 Paciente masculino, 57 anos, colidiu com outro ciclista durante uma competição e caiu sobre o glúteo direito contra uma pedra. Ele deu entrada no pronto-socorro com dor e edema importantes na região glútea direita. De acordo com o protocolo do Advanced Trauma Life Support (ATLS), o paciente estava com sua via aérea pérvia, respiração normal, com sinais de choque classe II (frequência cardíaca superior a 100 batimentos por minuto), apresentava escore 15 na escala de coma de Glasgow e sem sinais de instabilidade óssea pélvica. No exame físico, foi notado importante hematoma, não pulsátil, na região lombar e do glúteo direito, com dor à palpação local. O paciente também referia parestesia no pé direito, provavelmente causada por compressão do nervo ciático. Exames laboratoriais mostraram queda da hemoglobina (< 7 g/dL), iniciando-se a reposição com concentrado de hemácias. Foi realizada uma angiotomografia de abdome e pelve, a qual evidenciou hematoma no glúteo à direita com extravasamento de contraste, condizente com um pseudoaneurisma no local ( Figura 1 ). O paciente foi então encaminhado para a sala de hemodinâmica e submetido a angiografia, que confirmou um pseudoaneurisma da artéria glútea inferior ( Figura 2 ). 

**Figura 1:** Seta representa a imagem tomográfica da lesão com extravasamento de contraste em região glútea direita e hematoma associado.

**Figura 2:** Seta representa o aspecto angiográfico do pseudoaneurisma da artéria glútea inferior direita.

 Para a correção do pseudoaneurisma, foi realizada uma embolização superseletiva, ocluindo esse ramo pré e pós-lesão com molas de destaque controlado (da marca Codman & Shurtleff, Inc; da empresa Johnson & Johnson, Raynham, MA, EUA) para a interrupção completa do sangramento no mesmo momento da angiografia ( Figuras 3 3C). Após a embolização, o hematoma foi drenado para reduzir o risco de necrose glútea, diminuir a dor e melhorar os sinais e sintomas neurológicos. O paciente foi encaminhado para a unidade de terapia intensiva, onde foi realizado o controle dos parâmetros hemodinâmicos e laboratoriais e foi complementada a reposição volêmica. Houve melhora imediata da dor no glúteo e da parestesia. O paciente foi subsequentemente transferido para outro hospital, com o sangramento controlado. 

**Figura 3:** (A) Imagem angiográfica do intraoperatório evidenciando perviedade do pseudoaneurisma; (B e C) Aspecto angiográfico final após embolização com molas mostrando a completa oclusão do pseudoaneurisma.

## DISCUSSÃO

 As principais artérias que irrigam a região glútea são as artérias glúteas superior e inferior. A artéria glútea inferior é o maior ramo terminal do segmento anterior da artéria ilíaca interna. O pseudoaneurisma é causado por uma ruptura da parede do vaso, apresentando-se como um hematoma pulsátil que se comunica com a artéria. Normalmente, os ramos dessa artéria irrigam uma porção maior do glúteo em comparação aos ramos da artéria glútea superior 4 . Quando há uma queda contra o chão ou uma superfície rígida, ocasionando trauma contuso, a artéria glútea inferior e seus ramos têm potencial maior de lesão do que a artéria glútea superior devido ao trajeto que esses vasos percorrem na região glútea 1 ^,^ 3 . 

 Foram descritos menos de 150 casos de pseudoaneurismas de artéria glútea até o momento na literatura 5 . Mais comumente, essas lesões ocorrem na artéria glútea superior, relacionadas a traumas penetrantes, lesões iatrogênicas durante cirurgias pélvicas ou do quadril, fraturas dos ossos da pelve ou após injeções intramusculares 2 ^,^ 4 . Elas podem ocorrer logo depois do trauma inicial ou após semanas, meses ou anos do evento 3 . Menos frequentemente, são causadas por traumas fechados, como o aqui descrito. 

 Os pseudoaneurismas podem ser assintomáticos; porém, a apresentação mais comum é com edema e/ou hematoma doloroso na região glútea. Como os sintomas não são específicos, há a necessidade de ser feito o diagnóstico diferencial de abscesso glúteo ou de dor de origem ciática 4 . Os sinais mais comuns são hematoma pulsátil e sopro na região glútea acometida, que podem estar associados a inflamação local ou ainda a sintomas de compressão do nervo ciático 3 ^,^ 4 . Podem apresentar-se como choque hemorrágico, quando há rotura do pseudoaneurisma, configurando-se essa como a forma de apresentação mais grave. Um diagnóstico errôneo de abscesso local pode resultar em evolução catastrófica 1 ^,^ 3 ^,^ 4 . No caso descrito aqui, os sinais identificados foram hematoma doloroso e não pulsátil em glúteo direito. 

 Devido ao risco de uma situação potencialmente fatal, o diagnóstico precoce e o tratamento imediato são fundamentais. A angiotomografia tem grande acurácia para o diagnóstico do pseudoaneurisma e para diferenciá-lo de um abscesso ou tumoração de tecidos moles 1 ^,^ 5 ^,^ 6 . No presente caso, o paciente foi submetido a angiotomografia, pois encontrava-se estável hemodinamicamente. A ultrassonografia com Doppler pode ser o primeiro exame complementar a ser realizado na entrada do paciente, pois é de baixo custo e não invasivo, podendo revelar fluxo arterial no interior do hematoma e fazer a diferenciação de outras lesões em partes moles 1 ^,^ 5 . 

 Apesar de a angiografia permitir confirmar o sítio do pseudoaneurisma e se o tratamento endovascular imediato é factível, as lesões das artérias glúteas podem ser tratadas tanto pela abordagem cirúrgica convencional (Battle) quanto por técnicas endovasculares, que são minimamente invasivas, como as embolizações transcateter 7 ^,^ 8 . As vantagens da embolização incluem: ausência de cicatriz, redução do risco de infecção, preservação do espaço retroperitonial, menor risco de lesões iatrogênicas de nervo ou vasculares, e menor tempo de permanência no hospital 9 ^,^ 10 . Nesse tipo de caso, a embolização superseletiva é um método efetivo, seguro e confiável para a interrupção do sangramento arterial, especialmente na região pélvica 2 ^,^ 11 ^,^ 12 . No presente caso, a embolização foi realizada com sucesso, em regime de urgência, para interromper o sangramento e evitar o potencial dano permanente ao nervo ciático pela compressão causada pelo hematoma glúteo em expansão. O risco potencial desse tipo de procedimento (embolização) é a ocorrência de isquemia do músculo glúteo 2 ^,^ 13 ^,^ 14 . A realização de uma embolização superseletiva com molas de destaque controlado reduz esse risco 1 ^,^ 11 . Como alternativa à embolização com molas, pode-se realizar a embolização com cola ou outros materiais emboligênicos, como as colas de cianoacrilato e ônix 14 . 

 Como alternativa à embolização com molas, alguns autores também sugerem a injeção de trombina guiada por ultrassom no local da lesão. Essa é uma forma de tratamento amplamente utilizada em pseudoaneurismas superficiais e de colo estreito, como as lesões de artéria femoral comum pós-angiografia 1 ^,^ 15 . O risco desse procedimento é o possível refluxo da trombina para a circulação arterial, causando uma grave trombose dos vasos ao redor da lesão 4 . Neste caso, o pseudoaneurisma da artéria glútea era profundo e circundado pelo hematoma, o que dificultaria uma punção para injeção direta. 

## CONCLUSÃO

 O pseudoaneurisma da artéria glútea inferior ainda é uma afecção rara, de difícil diagnóstico e que desafia tanto o cirurgião do trauma quanto o cirurgião vascular. O tratamento endovascular é factível, seguro e eficaz, devendo ser a primeira escolha, quando viável, devido a sua menor invasividade quando comparado ao tratamento convencional.
